# Supplementary material for: Integrated Transcriptome and Metabolome Analysis Revealed the Causal Agent of Primary Bud Necrosis in ‘Summer Black’ Grape
Source: Int J Mol Sci. 2023 Jun 20;24(12):10410. doi: 10.3390/ijms241210410 (PMC10299459; doi:10.3390/ijms241210410)
Supplement: Supplementary file 1 [file ijms-24-10410-s001.zip › Supplementary Figures.pdf]

Integrated Transcriptome and Metabolome Analysis Revealed Primary bud Necrosis in ‘Summer Black’ Grape

Shaogang Fan, Yanshuai Xu, Miao Bai, Feixiong Luo, Jun Yu, Guoshun Yang*

College of Horticulture, Hunan Agricultural University, Changsha, Hunan 410128, PR China

Supplementary Figures List

Supplementary Figures S1. Principal component analysis of transcriptome data.

Supplementary Figure S2. The DEGs gene ontology functional classification

Supplementary Figure S3. Go enrich analysis of DEGs

Supplementary Figure S4. Categories of common DAMs in the comparison of BN vs. PN and BN vs. AN

Supplementary Figure S5. Clustering analysis of DAMs in the comparison of BN vs. PN and BN vs. AN.

Supplementary Figure S6. Co-enrichment analysis of DEMs and DEGs in KEGG pathways

Supplementary Figure S7. The Co-enriched KEGG pathways of correlation analysis of differentially accumulated metabolites and differentially expressed genes.

Supplementary Figures

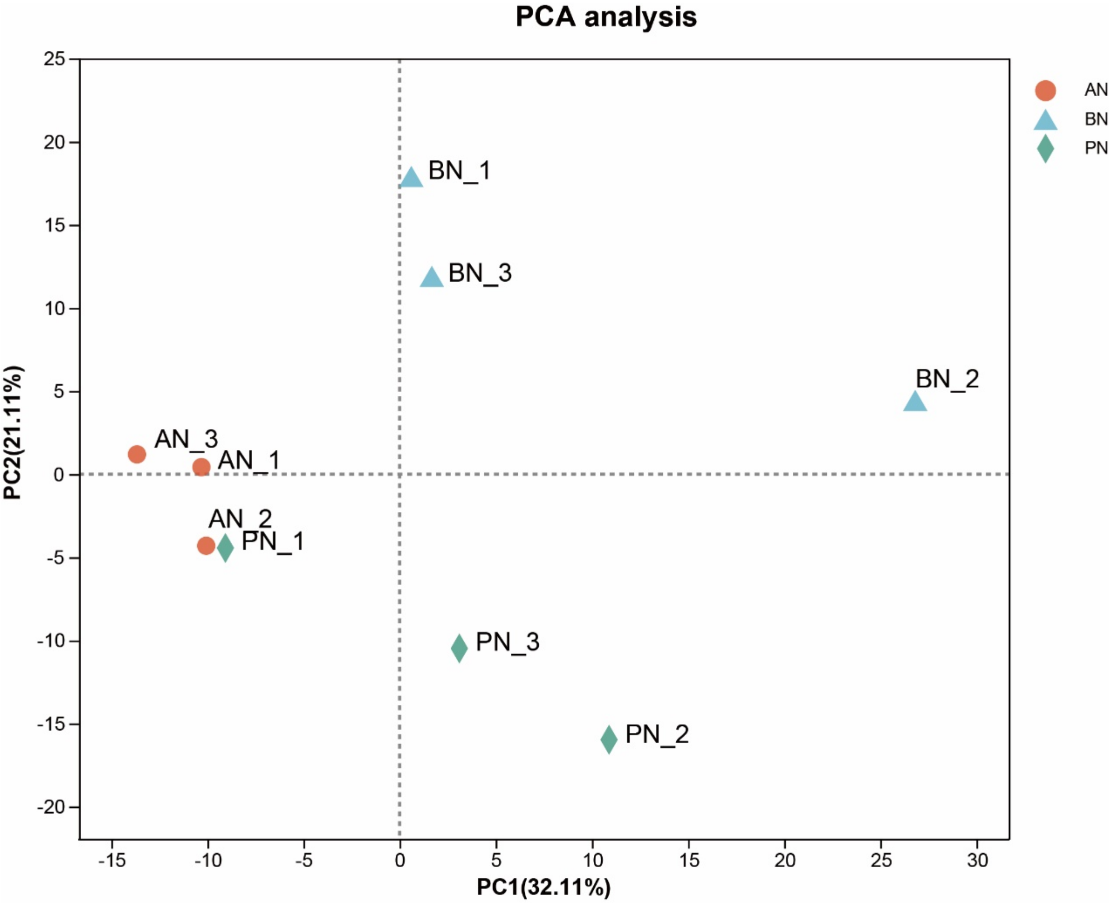

Figures S1. Principal component analysis of transcriptome data.

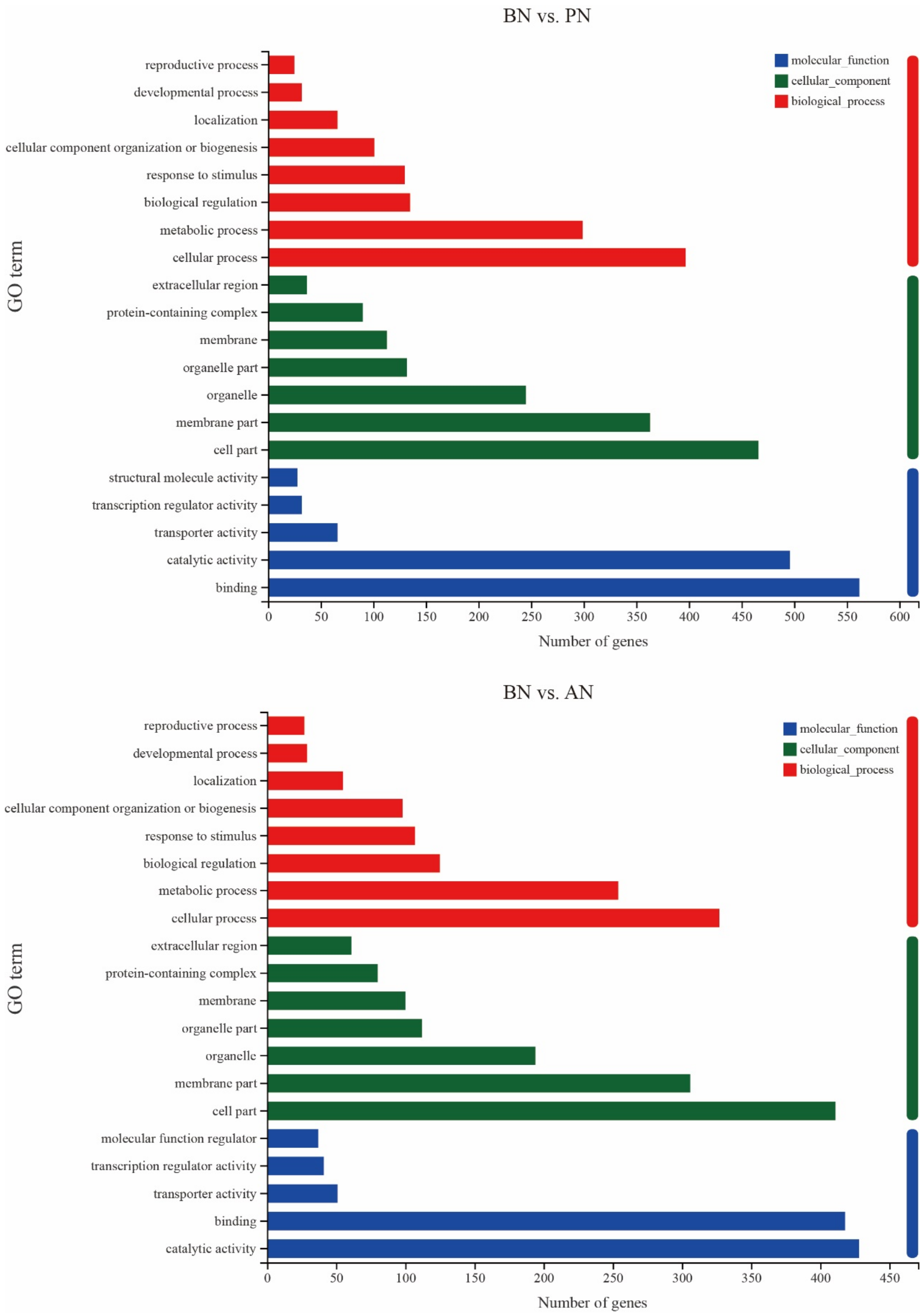

Figure S2. The DEGs gene ontology functional classification

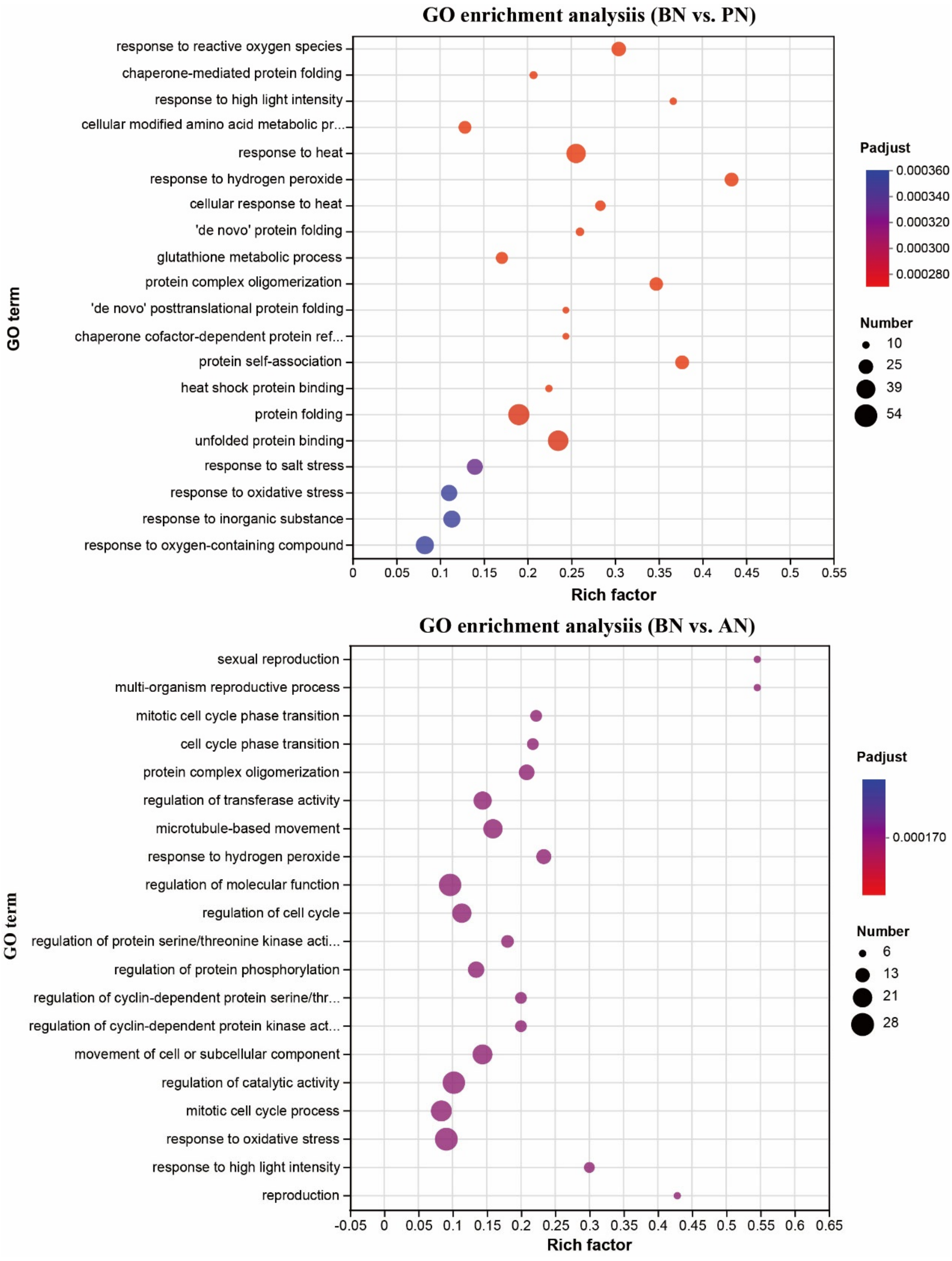

Figure S3. Go enrich analysis of DEGs

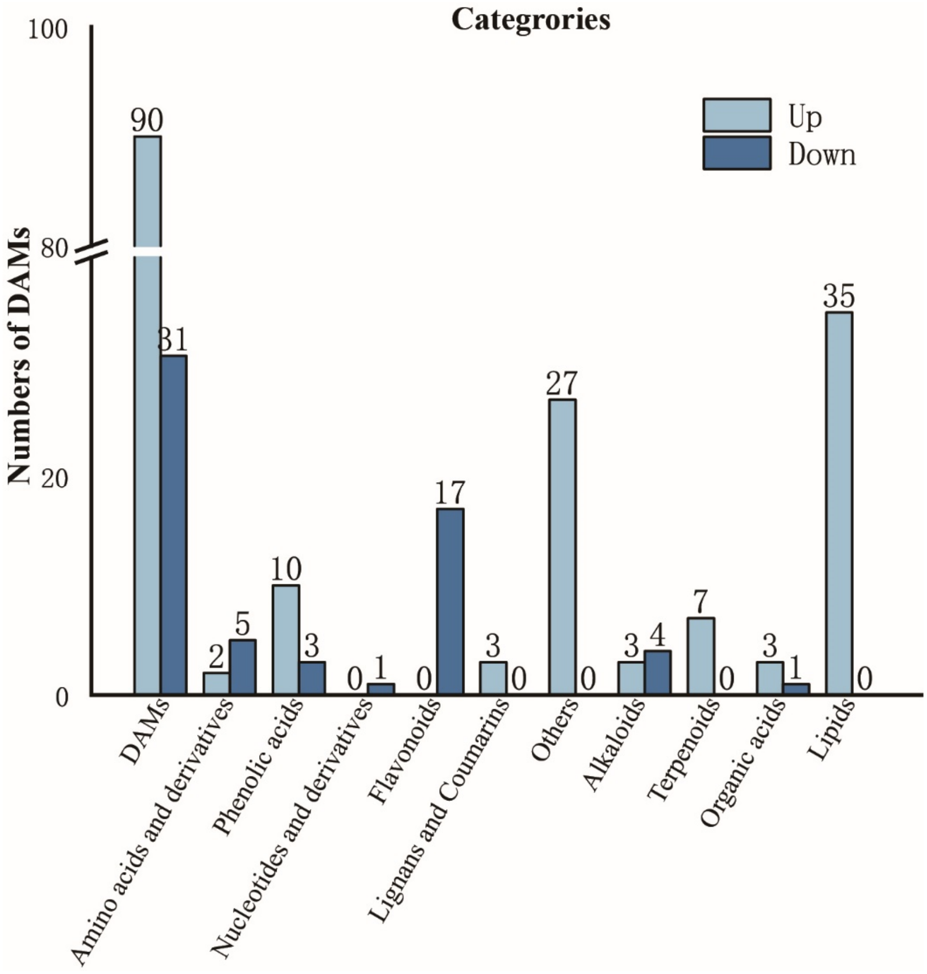

Figure S4. Categories of common DAMs in the comparison of BN vs. PN and BN vs. AN

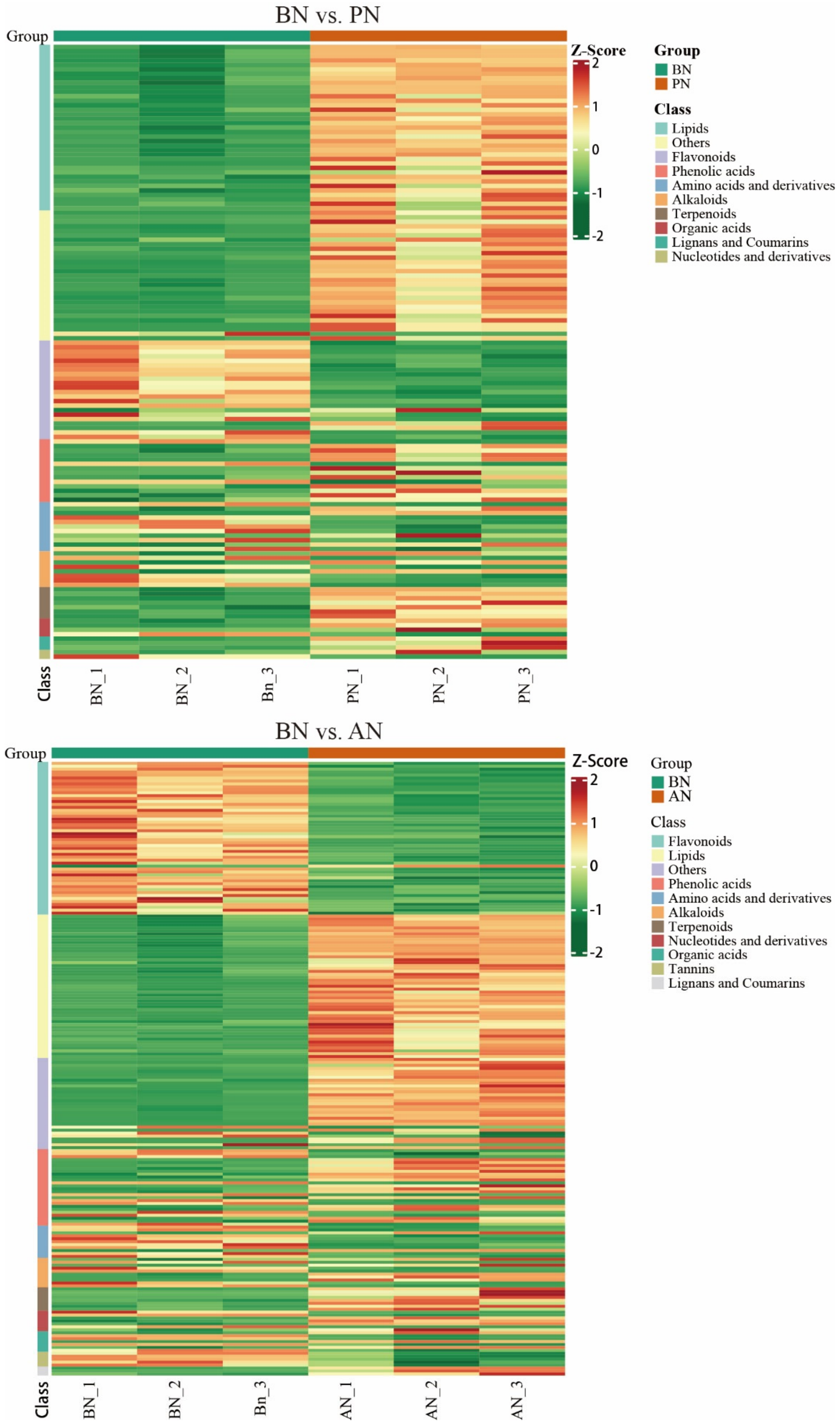

Figure S5. Clustering analysis of DAMs in the comparison of BN vs. PN and BN vs. AN

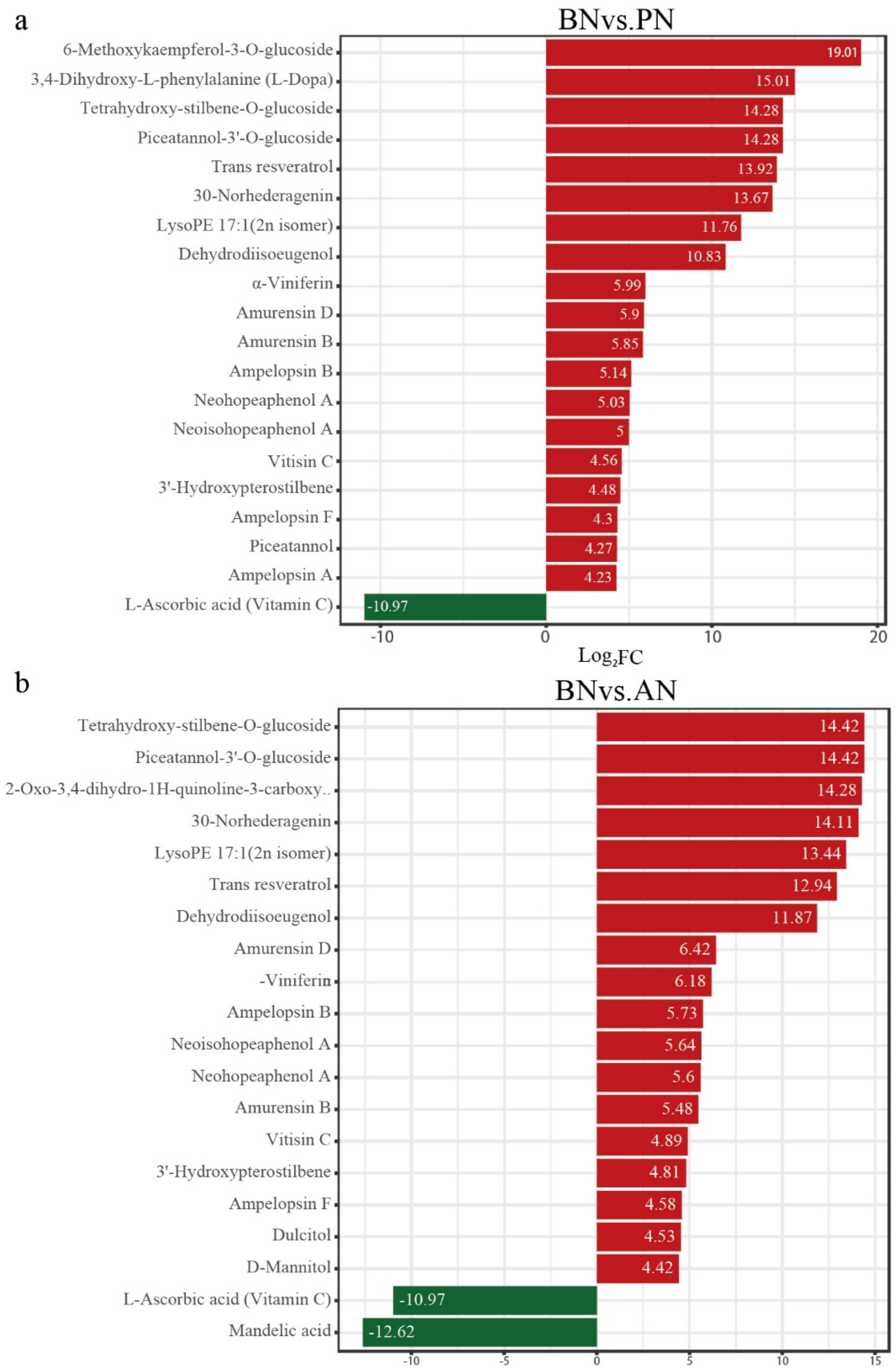

Figure S6. The bar graph of the top 20 differentially expressed metabolites.

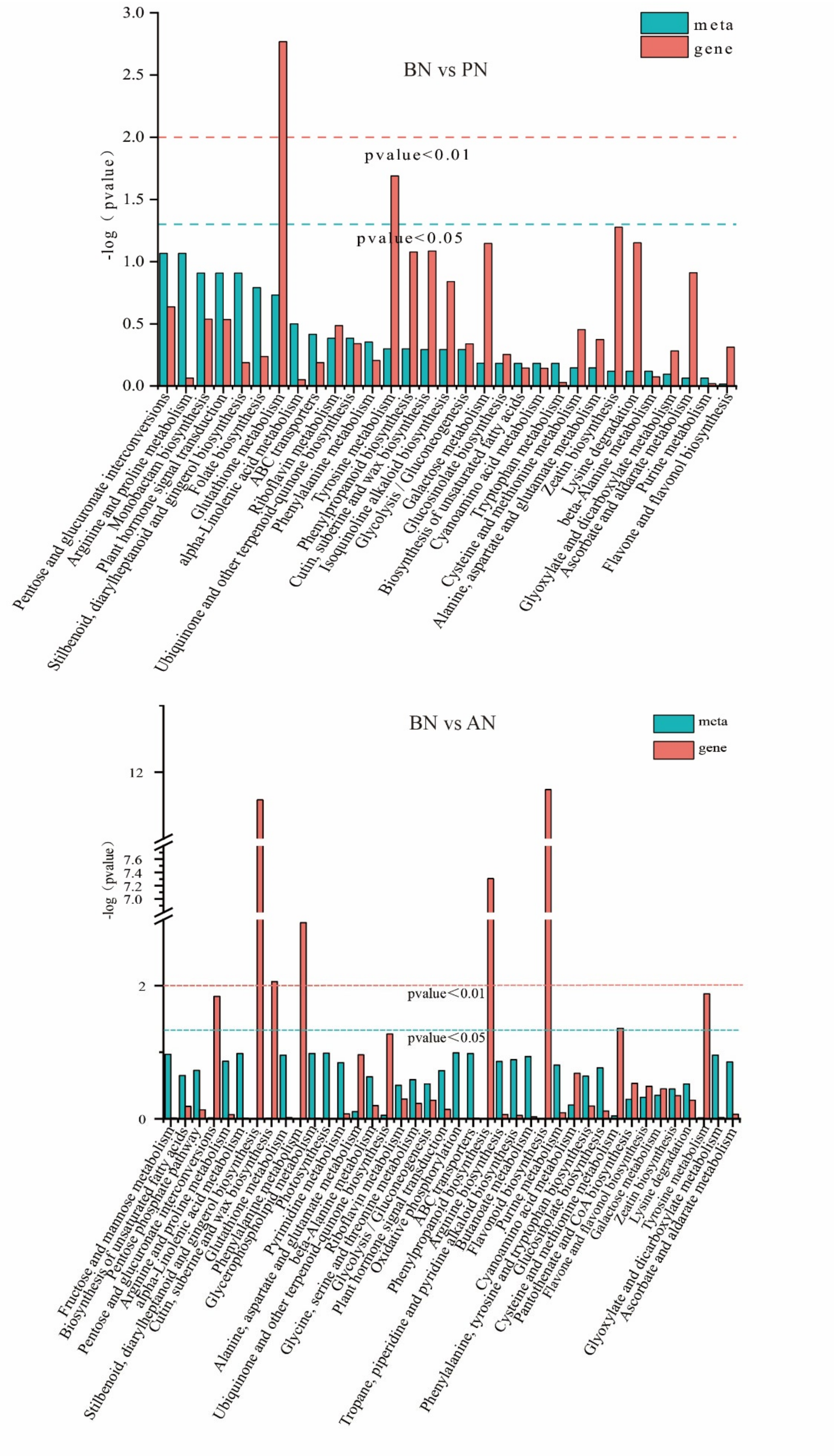

Figure S7. The Co-enriched KEGG pathways of correlation analysis of differentially accumulated metabolites and differentially expressed genes.
